# Supplementary material for: Discovery and application of insertion-deletion (INDEL) polymorphisms for QTL mapping of early life-history traits in Atlantic salmon
Source: BMC Genomics. 2010 Mar 8;11:156. doi: 10.1186/1471-2164-11-156 (PMC2838853; doi:10.1186/1471-2164-11-156)
Supplement: Additional file 2 — Information on developed 76 locus single-run INDEL panel in Atlantic salmon. Information on fluorescence labeling, primer concentrations, PCR pooling and links to alignments, INDEL motifs and GENESCAN (Burge and Karlin 1997) predictions of genes/exons are available in html format. [file 1471-2164-11-156-S2.ZIP › Additionalfile2/snpsummary18256.html]

```
Cluster 9048 Contig 1

prev  Summary    Contig List  next
```

Size of Consensus sequence = 1041

Number of sequences = 6

Minimum redundancy = 2

Key

A gi|117490221|gb|EG822438.1|EG822438 EST\_ssal\_evd\_24175 ssalevd thymus Salmo salar cDNA Salmo salar cDNA clone ssal\_evd\_531\_155\_fwd 3', mRNA sequence  
B gi|117447482|gb|EG779701.1|EG779701 EST\_ssal\_evd\_2209 ssalevd thymus Salmo salar cDNA Salmo salar cDNA clone ssal\_evd\_501\_218\_fwd 3', mRNA sequence  
C gi|117492770|gb|EG824987.1|EG824987 EST\_ssal\_evd\_17686 ssalevd thymus Salmo salar cDNA Salmo salar cDNA clone ssal\_evd\_522\_245\_fwd 3', mRNA sequence  
D gi|117490222|gb|EG822439.1|EG822439 EST\_ssal\_evd\_24176 ssalevd thymus Salmo salar cDNA Salmo salar cDNA clone ssal\_evd\_531\_155\_rev 5', mRNA sequence  
E gi|117492759|gb|EG824976.1|EG824976 EST\_ssal\_evd\_17685 ssalevd thymus Salmo salar cDNA Salmo salar cDNA clone ssal\_evd\_522\_245\_rev 5', mRNA sequence  
F gi|117447471|gb|EG779690.1|EG779690 EST\_ssal\_evd\_2208 ssalevd thymus Salmo salar cDNA Salmo salar cDNA clone ssal\_evd\_501\_218\_rev 5', mRNA sequence

4 SNPs detected

A B C D E F  cosegregation weighted

533 - G G - G G   3/4 75.00
534 - T T - T T   3/4 75.00
749 A C C A C C   3/4 75.00
779 T T G G G G   1/4 25.00
